# Assessing the bioactivity of cannabis extracts in larval zebrafish

**DOI:** 10.1186/s42238-021-00103-y

**Published:** 2021-10-01

**Authors:** Jessica Nixon, Hanan Abramovici, Ashley Cabecinha, Camilo Martinez-Farina, Joseph Hui, Lee Ellis

**Affiliations:** 1 Aquatic and Crop Resource Development, National Research Council of Canada, 1411 Oxford Street, Halifax, Nova Scotia B3H 3Z1 Canada; 2 Office of Cannabis Science and Surveillance, Controlled Substances and Cannabis Branch, Health Canada, Ottawa, Canada

**Keywords:** Zebrafish, Cannabis extracts, ∆-9 Tetrahydrocannabinol, Cannabidiol, Cannabinoids, Entourage effect

## Abstract

**Background:**

Whole-plant cannabis extracts are consumed by the public for medical and non-medical (“recreational”) purposes but are poorly researched compared to pure cannabinoids. There is emerging evidence that cannabis extracts comprising complex mixtures of cannabinoids may have different biological effects from that of pure cannabinoids. In the current study, we sought to assess the effect of whole-plant cannabis extracts produced from different chemotypes of cannabis on the normal behavior of zebrafish larvae.

**Methods:**

Three cannabis plant chemotypes were used in this study that contained either high amounts of THC, high amounts of CBD, high but equal amounts of THC and CBD, or low but equal amounts of THC and CBD. Following solvent extraction, liquid chromatography coupled to high-resolution mass spectrometry (LC-HRMS) was performed for the detection and quantitation of target cannabinoids. Larval zebrafish behavioral models were subsequently used to assess the effect of the four different whole-plant cannabis extracts on the normal larval behavior using the DanioVision behavioral tracking systems and software. To compare, changes in the behavior activity levels for 30 min periods were compared to controls using 2-way ANOVA with multiple comparisons followed by a Bonferroni post hoc test.

**Results:**

It was found that the whole-plant extracts that contained high levels of THC had similar effects on larval behavior, while the high CBD and low THC:CBD extracts produced distinct effects on normal larval behavior. Exposure of larvae to concentration-matched levels of THC and CBD found in the extracts revealed that a subset of the cannabis extracts tested had similar behavioral profiles to the pure cannabinoids while others did not.

**Conclusions:**

To our knowledge, this is the first study to test and compare the bioactivity of different whole-plant cannabis extracts in larval zebrafish. This work will provide a framework for future studies of distinct cannabis extracts and will be useful for comparing the bioactivity of extracts from different cannabis chemotypes as well as extracts made through various heating processes. It will also act as the first stage of assessment before testing the extracts against zebrafish models of toxicity and disease.

## Background

Cannabis produces numerous biologically active substances and is used both for medical and non-medical (“recreational”) purposes. While the individual substances found in cannabis have varying biological effects of their own, studies have also reported complex interactions between individual substances (Andre et al., 2016; McPartland & Russo, 2001). Synergistic interactions between individual substances in cannabis are known typically as the “entourage effect.” For example, an entourage effect has been described between ∆-9 tetrahydrocannabinol (THC) and cannabidiol (CBD), the main cannabinoids found in cannabis (Russo & Guy, 2006; Russo, 2011). However, these interactions are complex and may also result in the inhibition of their individual effects depending on a number of factors, such as the ratios or levels of each compound and whether the compounds are administered sequentially or simultaneously (Russo, 2011; Canada Go, 2018; Zuardi et al., 2012; Davis & Hatoum, 1983; Freeman et al., 2019a).

Zebrafish larvae have become a well-recognized, sought-after model and a rapid screening tool for testing the bioactivity of neuroactive compounds, including cannabinoids (Selderslaghs et al., 2010; Parng et al., 2007; Sipes et al., 2011; de Esch et al., 2012; Rennekamp & Peterson, 2015). Zebrafish larvae have a highly conserved endocannabinoid system and express cannabinoid receptors and neurotransmitters found in mammalian systems (Krug & Clark, 2015; Lam et al., 2006; Rodriguez-Martin et al., 2007; McPartland et al., 2007). Several of the models developed use larval behavior as a platform to assess the activity of cannabinoids (Akhtar et al., 2013; Boa-Amponsem et al., 2019; Ellis et al., 2018; Hasumi et al., 2020; Samarut et al., 2019; Chousidis et al., 2020; Carty et al., 2019). These pharmacologically relevant behavioral models use potential changes in locomotion as assays to measure both basal activity and standard responses to external stimuli (Ellis & Soanes, 2012; Ingebretson & Masino, 2013; Irons et al., 2013). For example, the light/dark preference test is based on larvae exhibiting an innate avoidance response to dark environments and is considered an anxiety-like behavior that can be used to assess the efficacy of anxiolytics (Maximino et al., 2010; Steenbergen et al., 2011).

We have previously developed a multimodal behavioral assay that measures both the changes in baseline activity and a light/dark stimulus response as a platform to assess the effect of acute exposure to THC and CBD (Achenbach et al., 2018). In this previous study, we demonstrated acute exposure to each compound produces a distinct pattern of larval behavior, with THC exposure leading to a general decrease in activity, while CBD decreased or eliminated the normal larval response to the light/dark transition at levels that did not affect the baseline activity. When combined, THC and CBD appeared to display an entourage effect on larval behavior. Additionally, we have shown that THC and CBD can oppose several larval behaviors generated by disease models of pain (nociception) and seizures (Ellis et al., 2018; Samarut et al., 2019). These studies substantiate the use of zebrafish larval behavior as a model for researching the effects of cannabinoids and provide a platform with which to test the bioactivity of other cannabinoids, cannabinoid products, and complex mixtures.

In the current study, we sought to assess the effect of whole-plant cannabis extracts produced from different chemotypes of cannabis on the normal behavior of zebrafish larvae. In order to assess if the acute larval behavioral response patterns produced by these different extracts were primarily due to the effects of THC and CBD, we also compared the activity patterns produced by the extracts to concentration-matched levels of THC and CBD in each extract. We found that each extract produced unique and partially overlapping effects on larval behavior. Importantly, we have also shown that exposure to THC and CBD alone is not sufficient to replicate the behavioral patterns produced by exposure to the extracts. These data raise the possibility that other extract components may modulate the observed effects on behavior.

## Materials and methods

### Fish husbandry

Adult zebrafish (*Danio rerio*) were maintained according to standard animal care protocols (Westerfield, 1995; Westerfield, 2000), in accordance with the Canadian Council of Animal Care (CCAC) guidelines. Adult AB/Tubingen zebrafish were housed on a re-circulating aquatic system at 28.5 ± 1 °C, pH 7.0–7.2 on a 14:10-h light:dark schedule. Embryos from multiple breeding pairs were collected and pooled in buffered E3 media (5 mM NaCl, 0.17 mM KCl, 0.33 mM CaCl_2_-2H_2_O, 0.33 mM MgSO_4_-7H_2_O, 10 mM HEPES, and pH 7.2) for 4–6 h. Following incubation at 28.5 ± 1 °C, embryos were housed in nursery baskets (< 200 embryos per basket) (Pentair Aquatic Eco-system, Apopka, FL, USA) in 3-L tanks (Tecniplast, Buguggiate, VA, Italy), with matching conditions to adults. Larvae were removed from the re-circulating system using buffered E3 media at 120 h post-fertilization (hpf) to be used in experiments that day.

### Plant extract generation

Three cannabis plant chemotypes, purchased from Aurora Cannabis (Vancouver, British Columbia, Canada), were used in this study to generate four distinct extracts. One chemotype contained high levels of THC (Henik, Aurora Cannabis), one contained high levels of CBD (Treasure Island, Aurora Cannabis), and the last chemotype contained equal amounts of THC and CBD (#385, Aurora Cannabis) albeit prepared two different ways to create two extracts with differing cannabinoid compositions. The four extracts were prepared from ground, dried cannabis plant material prepared with a coffee grinder. Samples were then weighed out in triplicate using approximately 200 mg of dried cannabis plant material for extraction. For three of the extracts, dried plant material was heated in a 95 °C temperature oven without solvent for 3 h to decarboxylate acid cannabinoids to their neutral forms. Following decarboxylation, 6 mL of pure 100% ethanol were added, and the sample was sonicated for 10 min at room temperature, the solvent was removed, and the sample was sonicated again with a fresh 6 mL of ethanol. Both ethanol fractions were then combined, filtered, dried, and weighed. The same extraction process as above was followed to make the fourth extract with the exception that no heating was applied to create the extract with low levels of both THC and CBD. The extracts were re-suspended in 2 mL of pure 100% methanol and diluted to a working stock concentration of 1 mg/mL in pure 100% methanol. The four extracts created are identified in this paper as high THC, high CBD, high-temp THC:CBD, and low-temp THC:CBD.

### Cannabinoid quantitation using LC-HRMS

Liquid chromatography coupled to high-resolution mass spectrometry (LC-HRMS) was performed on a mass spectrometer (Thermo Exactive™, Ottawa, Ontario, Canada), equipped with an electrospray ionization source. This allowed for the detection of target cannabinoids with a mass accuracy of less than 5 ppm. Separation was performed on an UPLC HSS-T3 column (2.1 × 100mm 1.8 μm, Acquity) (Waters, Milford, MA, USA) with gradient elution using mobile phases of 0.1% formic acid in de-ionized water and 0.1% formic acid in methanol. MS acquisitions were done in positive mode with instrument resolution at 50 K across a mass range from m/z 190–1000.

The 10 cannabinoid standards (pure THC and CBD were also used in the acute testing) were purchased from Sigma Aldrich (Darmstadt, Germany) and included CBD, CBDA, THC, THCA, CBC, CBCA, CBN, CBG, CBGA, and THCV (Cerilliant, Round Rock, TX, USA). The concentration of each standard was 1 mg/mL, and a dilution series containing all of these standards was prepared in methanol, at the following concentrations: 0.005 μg/mL, 0.01 μg/mL, 0.025 μg/mL, 0.05 μg/mL, 0.1 μg/mL, 0.25 μg/mL, 0.5 μg/mL, and 1 μg/mL. The extracts were diluted in methanol to ensure they fell within the linear range of the calibration curve to determine the amount of cannabinoids in extract materials (mg/g). We later presented the final concentration of the cannabinoids as a percentage of the total weight of the material.

### Acute behavioral testing

At 120 hpf, zebrafish larvae were transferred to a 48-well plate with 1 larva per well in 450 μL of buffered media using a micropipette and acclimated for 2 h at 28.5 °C in a light incubator. The extracts were stored as a 1 mg/mL 100% methanol stock solution at − 20 °C. Working solutions were prepared new each day at a 10× concentration in 0.5% methanol (also our control). Fifty microliters of 10× extract solution was pipetted into each well to reach the final concentration. In order to allow for a comparison between extracts, the dilution series tested was initially based on the weight concentration of each extract (μg/mL), and a standard dilution series was used for each compound (0.25–2 μg/mL). Each experimental replicate comprised 12 larvae per extract concentration which was completed in triplicate yielding a final 36 larvae per treatment and 96 for controls (combined over extract experiments). Following the addition of the extract solution, each plate was placed directly into the DanioVision-automated behavioral tracking system (Noldus, Wageningen, the Netherlands) and exposed to our standardized behavioral assay which consisted of 1.5 h of consistent white light (15–16 μM/s/m) followed by alternating 5-min periods of light or dark for 30 min. Following the behavioral assessment, the larvae were visually scored for any abnormalities, and dead, necrotic, or visually affected larvae were removed from the behavioral analysis. The patterns of behavior induced by each extract were assessed using the EthoVisionXT13/14/15 software (Noldus). Behavioral profiles were generated by averaging the total distance traveled (mm) quantified into 60-s bins. We then selected three periods of the 2-h experiment for further statistical analysis, the first 30 min of the experiment, the period from 60–89 min, and the third during the first light to dark transition. To quantify the changes in behavior during these periods, activity levels for the two 30-min periods were compared to controls using 2-way ANOVA with multiple comparisons followed by a Bonferroni post hoc test where *p* < 0.05. To quantify the response of a transition from a light to a dark environment, we normalized the total average activity (distance moved) in the first 5-min dark transition to the previous 5 min in the light and again used 2-way ANOVA with a Bonferroni post hoc (*p* < 0.05) test to determine the statistical significance (GraphPad, San Diego, CA, USA).

## Results

### Cannabinoid profile of extracts

Three separate cannabis chemotypes were obtained from a Canadian commercial supplier and were selected based on their varying cannabinoid compositions. The chemotypes are described based on their levels of THC and CBD with the first having high levels of THC and no CBD (high THC), the next had a high level of CBD and a low level of THC (high CBD), and the last had roughly equal amounts of THC and CBD (high-temp THC:CBD and low-temp THC:CBD). The equal ratio chemotype was used to create two extracts that contained primarily either decarboxylated or non-decarboxylated cannabinoids. The first was generated by exposing the plant material to a high temperature (high temp) before the extraction, and the second was left at room temperature (low temp) before the extraction (Table 1).

**Table 1:** Analysis of the average cannabinoid concentrations in extracts prepared from different cannabis chemotypes as measured by LC-HRMS (%). *ND* not detectable

Extract ID	Cannabinoid quantification (%)									
	CBD	THC	CBDA	THCA	CBC	CBCA	CBN	CBG	CBGA	THCV
High THC	ND	80.8	0.10	2.19	0.83	ND	0.60	1.22	0.57	0.56
High CBD	63.8	3.01	17.2	ND	3.48	0.61	ND	1.20	0.51	ND
Low-temp THC:CBD	1.31	2.41	29.9	21.8	ND	2.27	ND	ND	0.43	ND
High-temp THC:CBD	28.3	28.5	9.51	0.57	1.59	ND	0.14	0.44	0.30	ND

Each extract was profiled by LC-HRMS in order to measure the levels of the 10 primary cannabinoids with commercially available standards. The most notable differences in the cannabinoid profile were for the low-temp THC:CBD extract which had low levels of both THC and CBD and higher levels of THCA and CBDA than were present in the other extracts. In comparison with the other extracts evaluated, the high CBD extract had a higher level of CBC and both the high THC extract and the high CBD extracts had higher levels of CBG (Table 1).

### Acute effects of extracts on behavior

**Fig. 1:**
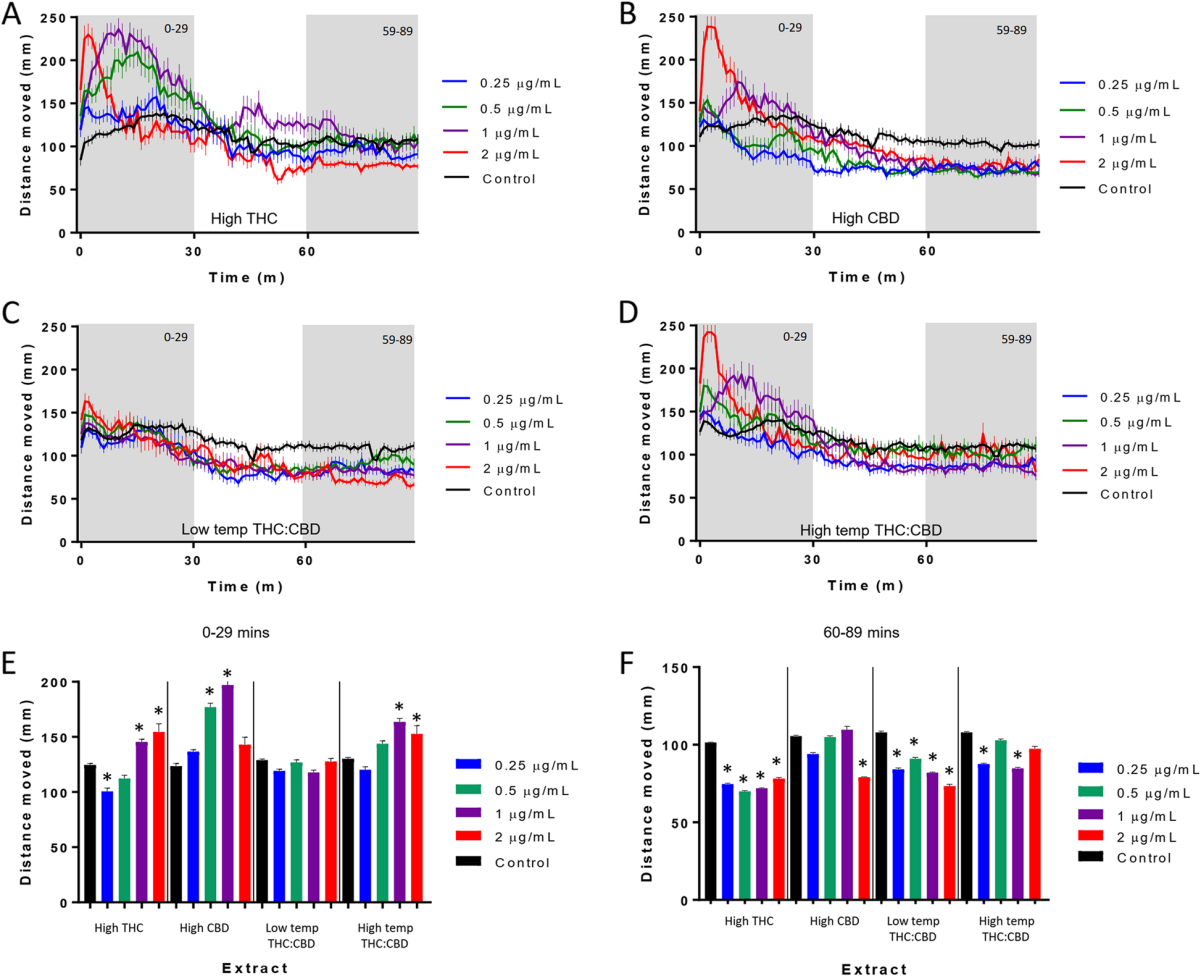
Larval locomotion following exposure to high THC, high CBD, low-temp THC:CBD, and high-temp THC:CBD extracts. **A**–**D** Larval behavioral profiles following exposure to extracts at varying concentrations. The average distance traveled is presented in millimeters per 1 min bin ± standard error (*SEM*) and time in minutes. **E**, **F** The average distance traveled ± *SEM* is presented in millimeters per 30 min bin and time in minutes. Each bar represents an *n* = 36 (extract) to 96 (control) larvae. *Significant difference from experimental control as measured by ANOVA followed by a Bonferroni post hoc test (*p* < 0.05)

The acute behavioral response of zebrafish larvae following exposure to individual extracts was used as a model to assay the potential neural activity of each extract. Larval behavior was initially monitored for 90 min in the light followed by three successive 5 min periods of alternating dark and light transitions, which induce a stress response (Maximino et al., 2010; Steenbergen et al., 2011). The activity pattern produced during the initial period of light was previously used to assess the effect of acute exposure to THC and CBD on larval behavior. The previous study revealed that acute exposure to both THC and CBD could lead to an initial increase in activity during the first 10–30 min of exposure that then dropped to a level at which it plateaued. The plateau level for THC was lower than that of non-treated controls, while the CBD plateau level returned to levels not significantly different than controls at the concentrations tested (Achenbach et al., 2018). In the current study, acute exposure to the extracts produced similar patterns of activity; however, there were distinct concentration-dependent differences between the extracts. The high THC extract showed a peak in activity above control levels at 1 and 2 μg/mL that then decayed to a plateau level below that of controls (Fig. 1A). Exposure to the high CBD extract produced a similar initial peak in activity at 2 μg/mL; however, as the concentration was decreased, this initial increase in activity was sustained for longer periods of time remaining elevated for the first 30 min following exposure at 0.5 and 1 μg/mL (Fig. 1B). Unlike the high THC extract, the high CBD extract plateaued at a level that was not significantly different than controls. The low-temp THC:CBD extract showed only a small initial increase in activity at 2 μg/mL that then plateaued at a level lower than controls (Fig. 1C). The high-temp THC:CBD extract produced concentration response profiles that appeared to mirror that of the high-THC extract (Fig. 1D). In order to make direct comparisons of the concentration response profiles between the extracts, we compared the total activity for the first 30 min following exposure and the final 30 min of the light cycle, which represents the plateau period of the activity pattern. During the initial 30 min, there was a reduction in activity following exposure to the high THC extract at 0.25 μg/mL, while there was no significant difference between the other three extracts and controls (Fig. 1E). At 0.5 μg/mL, the high CBD extract produced a significant increase in activity, while there was no significant change in activity between the other three extracts and controls. At 1 and 2 μg/mL, the high THC, high CBD, and high-temp THC:CBD extracts all led to significant increases in activity, while the low-temp THC:CBD extract did not produce a change in activity compared to controls. During the final 30 min of the light cycle, the high THC and low-temp THC:CBD extracts produced a significant decrease in activity compared to controls at all concentrations tested, while the high CBD extract only showed a reduction in activity at 2 μg/mL and the high-temp THC:CBD reduced the activity at 0.25 and 1 μg/mL (Fig. 1F).

**Fig. 2:**
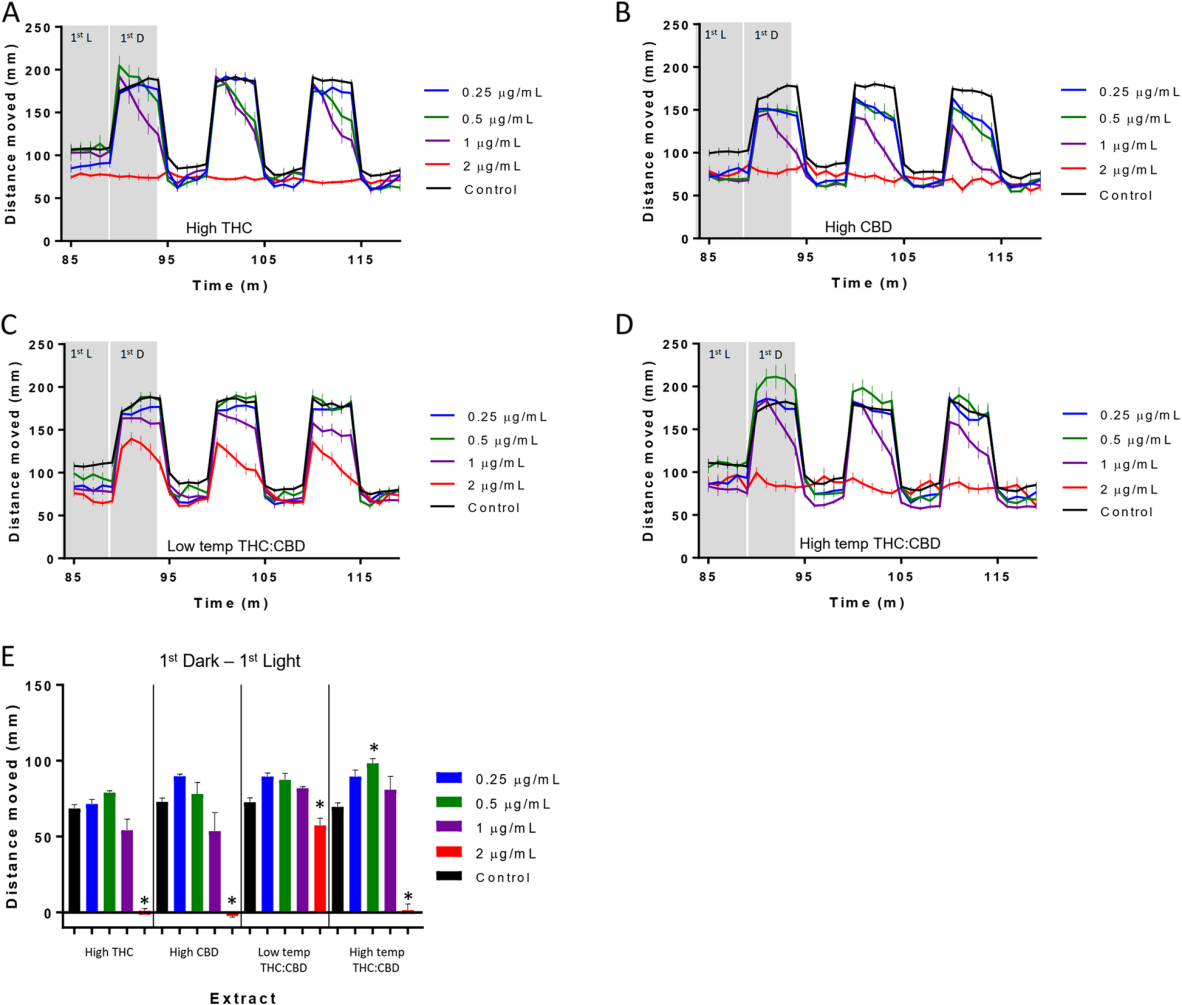
Larval locomotion following exposure to high THC, high CBD, low-temp THC:CBD, and high-temp THC:CBD extracts. **A**–**D** Larval behavioral profiles following exposure to extracts at varying concentrations during the alternating 5-min light/dark cycles. The average distance traveled is presented in millimeters per 1 min bin ± standard error (*SEM*) and time in minutes. **E** Difference in the average distance traveled between the first 5-min dark cycle and the preceding 5-min light. Average distance ± *SEM* is presented in millimeters per 5 min bin and time in minutes. Each bar represents an *n* = 36 (extract) to 96 (control) larvae. *Significant difference from experimental control as measured by ANOVA followed by a Bonferroni post hoc test (*p* < 0.05)

Following the 90 min in the light environment, the larvae were exposed to three successive light to dark transitions (Fig. 2). The transition from a light to a dark environment produces an increase in larval activity that is considered a stress response (Maximino et al., 2010; Steenbergen et al., 2011). Previous work from our lab showed distinct differences in the effect of acute exposure to THC or CBD on the light to dark transition response, with CBD reducing the response, while THC had a nominal effect on the light to dark transition at the concentrations tested (Achenbach et al., 2018). All four of the extracts tested showed a concentration-based reduction in the dark response with the high THC, high CBD, and high-temp THC:CBD extracts eliminating the response at 2 μg/mL (Fig. 2). In order to statistically compare the response to the transition, the activity during the 5 min in the light proceeding the dark transition was subtracted from the activity during the 5 min in the dark (Fig. 2E). It was found that the high THC and high CBD extracts reduced the response at 1 and 2 μg/mL, while the low temp THC:CBD reduced the response at 2 μg/mL. Interestingly, the high-temp THC:CBD mixture produced an increase in the dark response at 0.5 μg/mL and an elimination of the response at 2 μg/mL.

### Comparing the acute effects of matched concentrations of pure THC/CBD to those of the extracts

Since the changes in behavior produced by exposure to the extracts was similar to that previously shown for pure THC and CBD, we assessed if the behavioral response patterns produced by the different extracts were primarily due to the presence of THC and/or CBD. The larvae were exposed to concentration-matched levels of THC and CBD found in each extract at each of the extract concentrations tested above (Table 2).

**Table 2:** Calculated equivalent levels of pure THC and CBD (μM) found in the cannabis extracts at 0.25, 0.5, 1, and 2 μg/mL

Extract concentration (μg/mL)	0.25		0.5		1		2	
	CBD (μM)	THC (μM)	CBD (μM)	THC (μM)	CBD (μM)	THC (μM)	CBD (μM)	THC (μM)
**High THC**	0.00	0.64	0.00	1.29	0.00	2.57	0.00	5.14
**High CBD**	0.51	0.00	1.02	0.00	2.03	0.01	4.06	0.02
**Low-temp THC:CBD**	0.01	0.02	0.02	0.04	0.04	0.08	0.08	0.15
**High-temp THC:CBD**	0.23	0.23	0.45	0.45	0.90	0.91	1.80	1.81

**Fig. 3:**
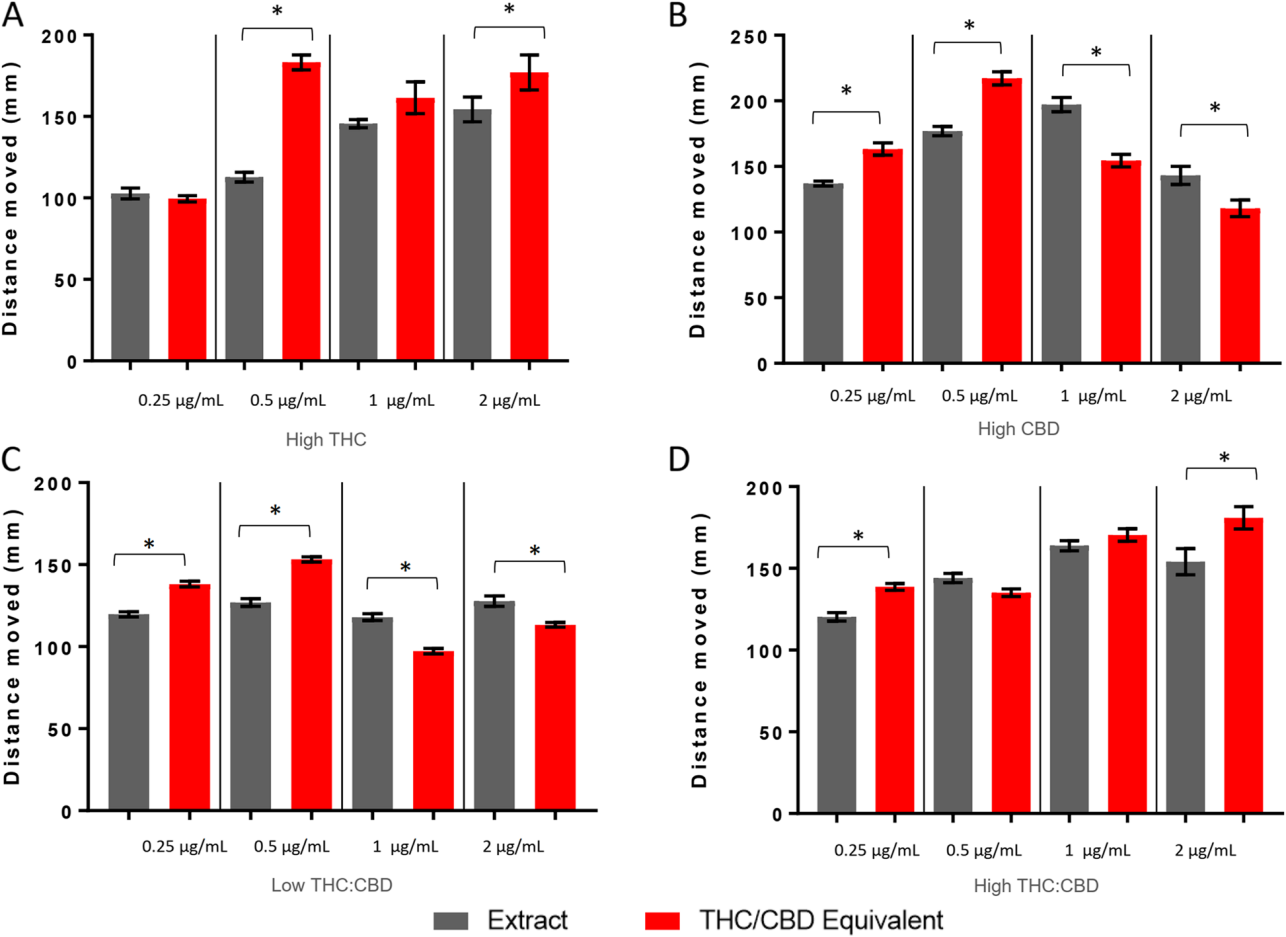
Larval locomotion following exposure to high THC, high CBD, low-temp THC:CBD, and high-temp THC:CBD extracts. Larval behavioral profiles following exposure to the extract and molar equivalence of THC/CBD at varying concentrations. Average distance traveled ± standard error (*SEM*) is presented over the first 30 min of the light cycle. Each bar represents an *n* = 36. *Significant difference from experimental control as measured by ANOVA followed by a Bonferroni post hoc test (*p* < 0.05)

During the first 30 min in the light following acute exposure, larvae treated with equimolar levels of THC/CBD to the high THC extract had higher activity levels than the extract at 0.5 and 2 μg/mL (Fig. 3A). For the high CBD extract and the low-temp THC:CBD extract, equimolar levels of pure THC/CBD produced higher levels of activity at 0.25 and 0.5 μg/mL and lower levels of activity at 1 and 2 μg/mL (Fig. 3B, C). Equimolar THC/CBD exposure led to an increased activity level compared to the high-temp THC:CBD extract at 0.25 and 2 μg/mL (Fig. 3D).

**Fig. 4:**
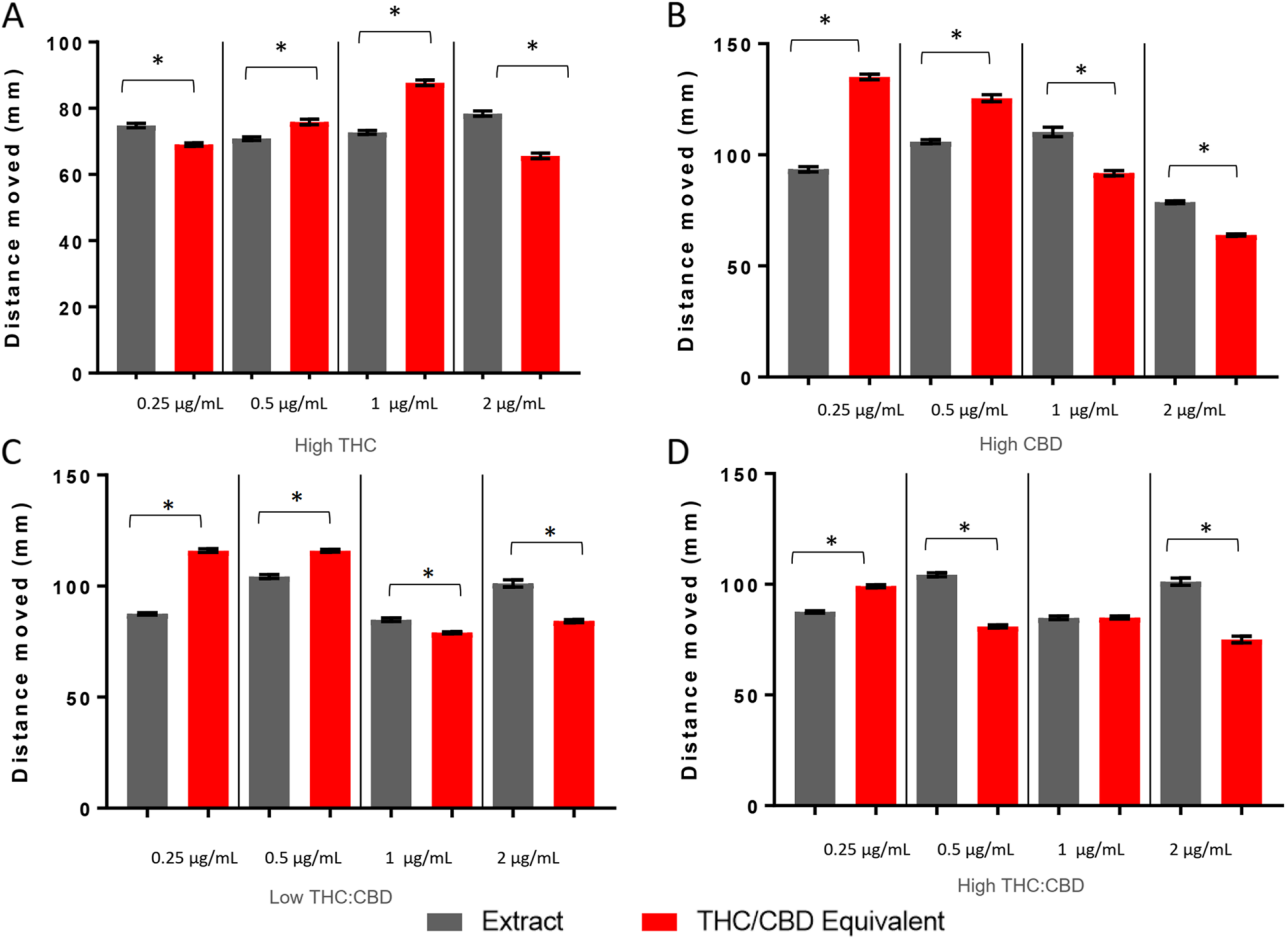
Larval locomotion following exposure to high THC, high CBD, low-temp THC:CBD, and high-temp THC:CBD extracts. Larval behavioral profiles following exposure to the extract and molar equivalence of THC/CBD at varying concentrations. The average distance traveled ± *SEM* is presented over 30 min (60–89 min) of the light cycle. Each bar represents an *n* = 36. *Significant difference from experimental control as measured by ANOVA followed by a Bonferroni post hoc test (*p* < 0.05)

For the plateau period, exposure to equimolar THC/CBD amounts produced a lower level of activity compared to the high THC extract at 0.25 and 2 μg/mL and a higher level of activity at 0.5 and 1 μg/mL (Fig. 4A). For the high CBD and low-temp THC:CBD extracts, exposure to equimolar THC/CBD produced a higher level of activity at 0.25 and 0.5 μg/mL and a lower level of activity at 1 and 2 μg/mL (Fig. 4B, C). Exposure to equimolar THC/CBD levels to the high temp THC:CBD resulted in a higher level of activity at 0.25 μg/mL and a lower activity level at 0.5 and 2 μg/mL (Fig. 4D).

**Fig. 5:**
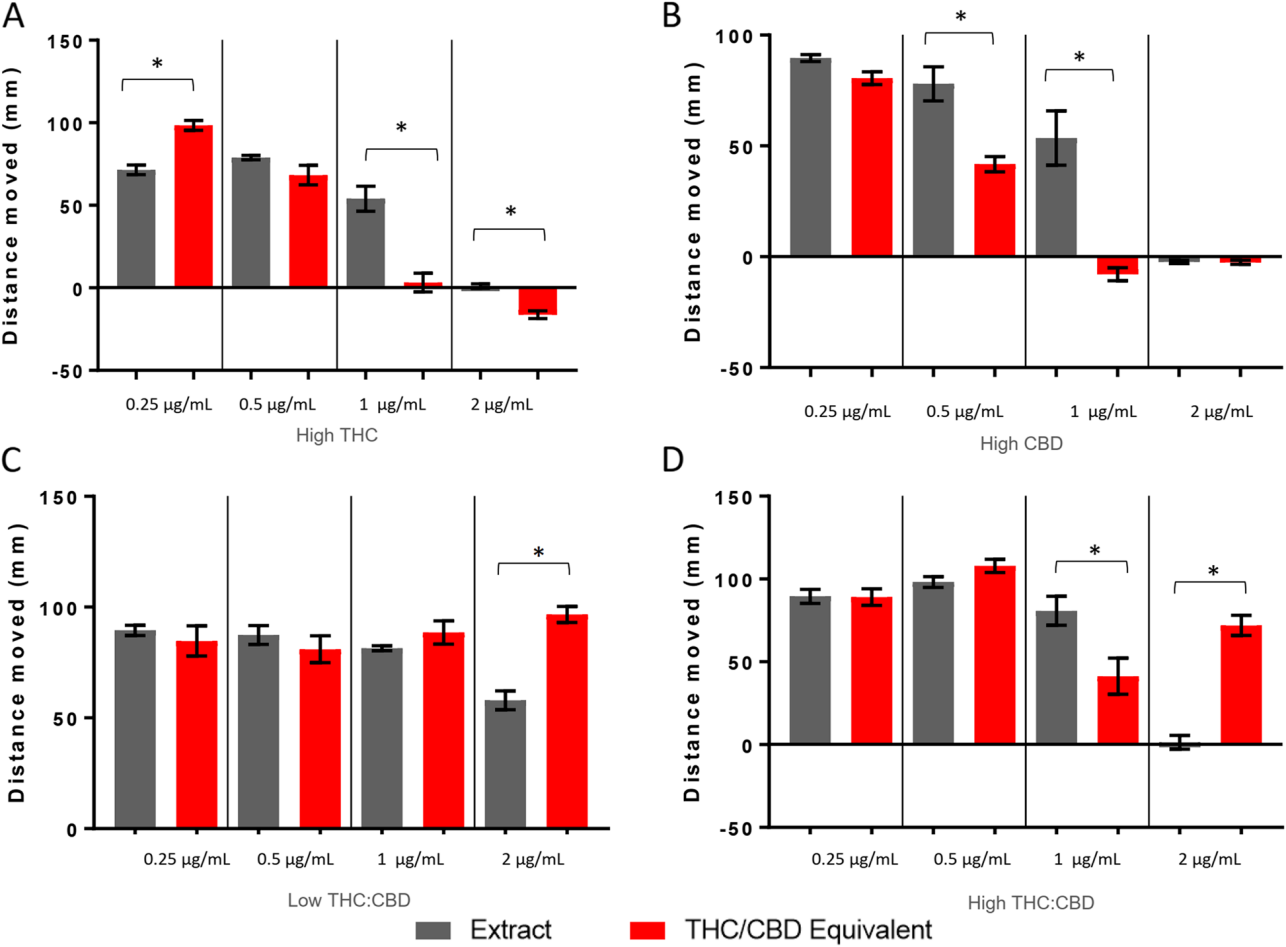
Larval locomotion following exposure to high THC, high CBD, low-temp THC:CBD, and high-temp THC:CBD extracts. Larval locomotion following exposure to extracts and equimolar THC/CBD equivalents at varying concentrations is presented as the difference in the average distance traveled between the first 5 min dark cycle and the preceding 5 min light ± *SEM*. Each bar represents an *n* = 36. *Significant difference from experimental control as measured by ANOVA followed by a Bonferroni post hoc test (*p* < 0.05)

Lastly, we compared the normalized activity levels for the dark transition. For the high THC extract, equimolar levels of THC/CBD produced a higher level of activity at 0.25 μg/mL and a lower level at 1 and 2 μg/mL (Fig. 5A). Exposure to the equimolar THC/CBD compared to the high CBD extract produced a lower activity level at 0.5 and 1 μg/mL (Fig. 5B). For the low-temp THC:CBD extract, equimolar THC/CBD exposure only showed a significant change in the activity level at 2 μg/mL where exposure to the equivalent THC/CBD amounts created a heightened response (Fig. 5C). The high-temp THC:CBD equimolar THC/CBD exposure produced a lower activity level at 1 μg/mL and a higher activity level at 2 μg/mL (Fig. 5D).

## Discussion

Numerous studies have shown that zebrafish have a highly homologous endocannabinoid system to that of humans and that behavioral models can be used to assess the bioactivity of the endocannabinoid system along with its potential as a therapeutic target (Krug & Clark, 2015; Ellis, 2018; Oltrabella et al., 2017). The previous studies have primarily tested pure compounds or mixtures thereof. The current study is the first to use zebrafish to test the behavioral effects associated with exposure to a series of different cannabis extracts. We have shown that this model of acute exposure in larval zebrafish is useful for identifying the distinct and concentration-dependent behavioral responses from exposure to cannabinoid extracts with unique cannabinoid profiles. We have also shown that extracts prepared from the same plant material by two different procedures (high-temperature and low-temperature exposure) display distinct acute effects on larval behavior.

Our lab has previously shown that larval exposure to pure THC or CBD resulted in distinct concentration-response behavioral profiles (Achenbach at al., 2018; Maximino et al., 2010). While both THC and CBD initially produced an increase in activity compared to controls, this increase was larger and more sustained following CBD exposure. In addition, following this increase in activity, the larvae exposed to THC had their activity level plateau at a level below controls, while the larvae exposed to CBD had their activity plateau at a level not significantly different than controls (Achenbach et al., 2018). The patterns of activity produced by the extracts in the current study appear to show similar complex concentration-dependent behavioral activity patterns compared to the pure THC and CBD profiles from the previous study. Not surprisingly, the high THC extract produced an activity pattern similar to pure THC, and the high CBD extract induced activity pattern was similar to pure CBD. Interestingly, the high-temp THC:CBD extract produced an activity pattern that appeared more aligned to that of the high THC rather than the high CBD extract.

In order to make a direct comparison between the activity of the pure cannabinoids and the extracts, the second part of the study compared the behavioral response following exposure to equivalent molar levels of pure THC and CBD to that found in each extract. Importantly, the activity patterns produced by exposure to pure THC and CBD did not consistently match that of the corresponding cannabis extracts themselves. The most pronounced difference was for the light-dark transition response where the high THC extract and the high CBD extract reduced, but did not eliminate the response to the dark stimulus at 1 μg/mL of extract, while the pure THC and CBD preparations at equimolar levels nearly eliminated the response. Similarly, exposure to the equimolar levels of THC/CBD to the high-temperature THC:CBD extract at 1 μg/mL reduced the transition response compared to the extract; however, in contrast to the high THC and high CBD extracts, at 2 μg/mL, the pure THC/CBD 1:1 treated larvae showed a higher transition response compared to the extract-treated larvae. Our aforementioned work has shown that, when combined, CBD can shift the concentration response pattern of THC at levels of CBD that are sub-phenotypic on their own (Achenbach et al., 2018). Consistent with these previous findings, our current results suggest that while THC and CBD are the dominant neuroactive compounds in the extracts tested, the activity of the other cannabinoids found in those extracts appear to be contributing to the differences in the activity levels of the extracts versus pure THC and CBD. This could be considered a more complex entourage effect where other components of the cannabis extracts (other cannabinoids, terpenes, etc.) act synergistically with THC and CBD to impact their activity (McPartland & Russo, 2001; Russo & Guy, 2006; Russo, 2011).

From our profiling of the four extracts, there appear to be different levels in each extract of the eight other cannabinoids evaluated. Some of the cannabinoids measured, specifically CBC, CBG, and CBDA, appear to be at concentrations that could contribute to the biological effect of the extracts on the larvae (Table 1). While THC and CBD interactions are well studied compared to other cannabinoids (Freeman et al., 2019b), there is growing evidence in mammalian models that other cannabinoids like the ones found in the extracts tested in this study can also impact the effects of THC. For example, THCV was subjectively noted to reduce the intensity of some effects caused by THC (e.g., dampening of the stereotypic increased heart rate associated with acute THC consumption) that may indicate a reduction in the potential side effects associated with THC exposure (Englund et al., 2015). In addition, co-administration of CBC with THC reduced THC-associated lethality in mice (Hatoum et al., 1981) and CBN has been observed to work synergistically with THC to potentiate the anti-nociceptive effects of THC (Welburn et al., 1976).

The mechanism by which the compounds found within the extracts may interact is complex. This is highlighted by the fact that CBD has been shown to antagonize the CB1 receptors in the presence of THC, while having little binding affinity for the CB1 receptor on its own (Thomas et al., 2007). In addition, CBD is known to have numerous potential target proteins outside of the CB1 receptor that may have an effect on the THC-induced larval activity and suggests a complex interaction with and effect on THC activity (Vitale et al., 2021). The complexity of the THC-CBD interaction is further increased by the presence of other cannabinoids and neuroactive compounds in the extracts. Future work will be required to study the effects that the other individual cannabinoids profiled in this study may have on larval behavior. This along with the study of complex mixtures of the pure compounds may help to ascertain the contributions of each to the changes in behavior and the mechanisms by which they interact.

Further chemical characterization of the extracts will also provide information on the presence of other potentially bioactive compounds found in cannabis such as terpenes, which may also exert biological effects.

## Conclusions

The importance of this study lies in the fact that we have now shown that zebrafish larvae can be used as a platform to assess the bioactivity of cannabis extracts. Moreover, our current acute model proves that these extracts have a distinct impact on baseline larval activity as well as stress responses in larvae. Cannabis extracts are a wide family of products that are used for both medical and non-medical (“recreational”) purposes. The chemical profile of the various extracts depends on the source of the material being extracted (i.e., “chemotype”) and the extraction solvent/method (Brighenti et al., 2017; Baranauskaite et al., 2020). We have shown in this study that a simple difference at a single step in the extraction procedure (i.e., temperature) can have a profound effect on the chemical profile and subsequent bioactivity of the extracts. While products derived from cannabis, such as extracts, are available for consumption by the public, there is currently a lack of knowledge of their effects. There is then an urgent need to study these products and to understand what biological effects these complex chemical mixtures may have. The results of this study provide a first step in using zebrafish models to further characterize these products and the interactions of the complex mixtures of chemicals found therein. Further work using the zebrafish model presented here along with other zebrafish larval models, such as various disease and toxicological models (Ellis et al., 2018; Samarut et al., 2019; Ellis & Soanes, 2012), may provide important insights into the safety and efficacy of using different cannabis extract-based products.

## Data Availability

Data and materials are available upon request to the corresponding author.

## Abbreviations

CCAC: Canadian Council of Animal Care
NaCl: Sodium chloride
KCl: Potassium chloride
CaCl_2_-2H_2_O: Calcium chloride dihydrate
MgSO_4_-7H_2_O: Magnesium sulfide heptahydrate
HEPES: 4-(2-Hydroxyethyl)-1-piperazineethanesulfonic acid
hpf: Hours post-fertilization
LC-HRMS: Liquid chromatography coupled to high-resolution mass spectrometry
THC: ∆-9 Tetrahydrocannabinol
CBD: Cannabidiol
CBDA: Cannabidiolic acid
THCA: Tetrahydrocannabinolic acid
CBC: Cannabichromene
CBCA: Cannabichromenic acid
CBN: Cannabinol
CBG: Cannabigerol
CBGA: Cannabigerolic acid
THCV: Tetrahydrocannabivarin
ANOVA: Analysis of variance

## References

[CR1] Achenbach JC, Hill J, Hui JPM, Morash MG, Berrue F, Ellis LD (2018). Analysis of the uptake, metabolism, and behavioral effects of cannabinoids on zebrafish larvae. Zebrafish.

[CR2] Akhtar MT, Ali S, Rashidi H, van der Kooy F, Verpoorte R, Richardson MK (2013). Developmental effects of cannabinoids on zebrafish larvae. Zebrafish.

[CR3] Andre CM, Hausman J-F, Guerriero G (2016). Cannabis sativa: the plant of the thousand and one molecules. Front Plant Sci.

[CR4] Baranauskaite J, Marksa M, Ivanauskas L, Vitkevicius K, Liaudanskas M, Skyrius V (2020). Development of extraction technique and GC/FID method for the analysis of cannabinoids in Cannabis sativa L. spp. santicha (hemp). Phytochem Anal.

[CR5] Boa-Amponsem O, Zhang C, Mukhopadhyay S, Ardrey I, Cole GJ (2019). Ethanol and cannabinoids interact to alter behavior in a zebrafish fetal alcohol spectrum disorder model. Birth Defects Res.

[CR6] Brighenti V, Pellati F, Steinbach M, Maran D, Benvenuti S (2017). Development of a new extraction technique and HPLC method for the analysis of non-psychoactive cannabinoids in fibre-type Cannabis sativa L.(hemp). J Pharm Biomed Anal.

[CR7] Canada Go (2018) Information for health care professionals: cannabis (marihuana, marijuana) and the cannabinoids. https://www.canada.ca/en/health-canada/services/drugs-medication/Cannabis/information-medical-practitioners/information-health-care-professionals-Cannabis-cannabinoids.html. 2021.

[CR8] Carty DR, Miller ZS, Thornton C, Pandelides Z, Kutchma ML, Willett KL (2019). Multigenerational consequences of early-life cannabinoid exposure in zebrafish. Toxicol Appl Pharmacol.

[CR9] Chousidis I, Chatzimitakos T, Leonardos D, Filiou MD, Stalikas CD, Leonardos ID (2020). Cannabinol in the spotlight: toxicometabolomic study and behavioral analysis of zebrafish embryos exposed to the unknown cannabinoid. Chemosphere.

[CR10] Davis WM, Hatoum NS (1983). Neurobehavioral actions of cannabichromene and interactions with delta 9-tetrahydrocannabinol. Gen Pharmacol.

[CR11] de Esch C, Slieker R, Wolterbeek A, Woutersen R, de Groot D (2012). Zebrafish as potential model for developmental neurotoxicity testing: a mini review. Neurotoxicol Teratol.

[CR12] Ellis L (2018). Zebrafish as a high-throughput in vivo model for testing the bioactivity of cannabinoids.

[CR13] Ellis L, Berrue F, Morash M, Achenbach J, Hill J, McDougall J (2018). Comparison of cannabinoids with known analgesics using a novel high throughput zebrafish larval model of nociception. Behav Brain Res.

[CR14] Ellis LD, Soanes KH (2012). A larval zebrafish model of bipolar disorder as a screening platform for neuro-therapeutics. Behav Brain Res.

[CR15] Englund A, Atakan Z, Kralj A, Tunstall N, Murray R, Morrison P (2015). The effect of five day dosing with THCV on THC-induced cognitive, psychological and physiological effects in healthy male human volunteers: a placebo-controlled, double-blind, crossover pilot trial. J Psychopharmacol.

[CR16] Freeman AM, Petrilli K, Lees R, Hindocha C, Mokrysz C, Curran HV (2019). How does cannabidiol (CBD) influence the acute effects of delta-9-tetrahydrocannabinol (THC) in humans? A systematic review. Neurosci Biobehav Rev.

[CR17] Freeman AM, Petrilli K, Lees R, Hindocha C, Mokrysz C, Curran HV (2019). How does cannabidiol (CBD) influence the acute effects of delta-9-tetrahydrocannabinol (THC) in humans? A systematic review. Neurosci Biobehav Rev.

[CR18] Hasumi A, Maeda H, Yoshida K-I (2020). Analyzing cannabinoid-induced abnormal behavior in a zebrafish model. PLoS One.

[CR19] Hatoum NS, Davis WM, Elsohly MA, Turner CE (1981). Cannabichromene and Δ9-tetrahydrocannabinol: interactions relative to lethality, hypothermia and hexobarbital hypnosis. Gen Pharmacol Vascul Syst.

[CR20] Ingebretson J, Masino M. Quantification of locomotor activity in larval zebrafish: considerations for the design of high-throughput behavioral studies. Front Neural Circuits. 2013;7:1–9. 10.3389/fncir.2013.00109 PMC3677137 23772207

[CR21] Irons TD, Kelly PE, Hunter DL, MacPhail RC, Padilla S (2013). Acute administration of dopaminergic drugs has differential effects on locomotion in larval zebrafish. Pharmacol Biochem Behav.

[CR22] Krug RG, Clark KJ (2015). Elucidating cannabinoid biology in zebrafish (Danio rerio). Gene.

[CR23] Lam CS, Rastegar S, Strähle U (2006). Distribution of cannabinoid receptor 1 in the CNS of zebrafish. Neuroscience.

[CR24] Maximino C, De Brito TM, de Mattos Dias CAG, Gouveia A, Morato S (2010). Scototaxis as anxiety-like behavior in fish. Nat Protoc.

[CR25] McPartland JM, Glass M, Matias I, Norris RW, Kilpatrick CW (2007). A shifted repertoire of endocannabinoid genes in the zebrafish (Danio rerio). Mol Genet Genom.

[CR26] McPartland JM, Russo EB (2001). Cannabis and cannabis extracts. J Cannabis Therapeut.

[CR27] Oltrabella F, Melgoza A, Nguyen B, Guo S. Role of the endocannabinoid system in vertebrates: emphasis on the zebrafish model. Development. 2017;59. 10.1111/dgd.12351 PMC5636690 28516445

[CR28] Parng C, Roy NM, Ton C, Lin Y, McGrath P (2007). Neurotoxicity assessment using zebrafish. J Pharmacol Toxicol Methods.

[CR29] Rennekamp AJ, Peterson RT (2015). 15 years of zebrafish chemical screening. Curr Opin Chem Biol.

[CR30] Rodriguez-Martin I, Herrero-Turrion MJ, Marron Fdez de Velasco E, Gonzalez-Sarmiento R, Rodriguez RE (2007). Characterization of two duplicate zebrafish Cb2-like cannabinoid receptors. Gene.

[CR31] Russo E, Guy GW (2006). A tale of two cannabinoids: the therapeutic rationale for combining tetrahydrocannabinol and cannabidiol. Med Hypotheses.

[CR32] Russo EB (2011). Taming THC: potential cannabis synergy and phytocannabinoid-terpenoid entourage effects. Br J Pharmacol.

[CR33] Samarut E, Nixon J, Kundap UP, Drapeau P, Ellis LD (2019). Single and synergistic effects of cannabidiol and delta-9-tetrahydrocannabinol on zebrafish models of neuro-hyperactivity. Front Pharmacol.

[CR34] Selderslaghs IW, Hooyberghs J, De Coen W, Witters HE (2010). Locomotor activity in zebrafish embryos: a new method to assess developmental neurotoxicity. Neurotoxicol Teratol.

[CR35] Sipes NS, Padilla S, Knudsen TB (2011). Zebrafish—as an integrative model for twenty-first century toxicity testing. Birth Defects Res Part C: Embryo Today: Rev.

[CR36] Steenbergen PJ, Richardson MK, Champagne DL (2011). Patterns of avoidance behaviours in the light/dark preference test in young juvenile zebrafish: a pharmacological study. Behav Brain Res.

[CR37] Thomas A, Baillie GL, Phillips AM, Razdan RK, Ross RA, Pertwee RG (2007). Cannabidiol displays unexpectedly high potency as an antagonist of CB1 and CB2 receptor agonists in vitro. Br J Pharmacol.

[CR38] Vitale RM, Iannotti FA, Amodeo P. The (poly)pharmacology of cannabidiol in neurological and neuropsychiatric disorders: molecular mechanisms and targets. Int J Mol Sci. 2021;22. 10.3390/ijms22094876 PMC8124847 34062987

[CR39] Welburn PJ, Starmer GA, Chesher GB, Jackson DM (1976). Effect of cannabinoids on the abdominal constriction response in mice: within cannabinoid interactions. Psychopharmacologia.

[CR40] Westerfield M (1995). The zebrafish book: a guide for the laboratory use of zebrafish (Brachydanio rerio).

[CR41] Westerfield M (2000). The zebrafish book: a guide for the laboratory use of zebrafish.

[CR42] Zuardi AW, Hallak JEC, Crippa JAS (2012). Interaction between cannabidiol (CBD) and ∆9-tetrahydrocannabinol (THC): influence of administration interval and dose ratio between the cannabinoids. Psychopharmacology.

